# Variation in Fatty Acid Synthase, Ki67 and p53 Esophageal Mucosa Expressions in Barrett’s Esophagus Patients Treated for One Year with Two Esomeprazole Different Regimens

**DOI:** 10.3390/cimb45060299

**Published:** 2023-05-29

**Authors:** Pietro Crispino, Tiziana Ciarambino, Mauro Giordano

**Affiliations:** 1 UOC of Internal Medicine, Santa Maria Goretti Hospital, 04100 Latina, Italy; 2 UOC of Internal Medicine, Hospital of Marcianise, “Caserta Local Health Authority”, 81025 Marcianise, Italy; 3 Advanced Medical and Surgical Sciences Department, University of Campania, L. Vanvitelli, 81100 Naples, Italy

**Keywords:** biological markers, esomeprazole, Barrett’s esophagus

## Abstract

Barrett’s esophagus (BE) is an acquired pre-malignant condition that results from chronic gastroesophageal reflux. The malignant transformation occurred in 0.5% of patients/year and was independent of medical and endoscopic conservative treatments. Fatty acid synthase (FAS) is a multifunctional enzyme that catalyzes the synthesis of long-chain fatty acids from acetyl-coenzyme A, malonyl-coenzyme A, a reduced form of nicotinamide adenine dinucleotide phosphate (NADPH), and adenosine triphosphate. Activation of FAS is closely linked to malignant transformation. The aim of the present study was to evaluate the variation of FAS, p53, and Ki67 expressions in two groups of 21 BE patients each, after one year of continuous (group A) or discontinuous (group B) treatment with esomeprazole 40 mg/day in comparison to the initial expression. In both the two groups of BE patients, biopsies were taken from pathologic sites of the mucosa for histological and immuno-histochemical detection of FAS, Ki67, and p53 at entry and after one year of Esomeprazole 40 mg treatment. FAS expression was positive when a strong granular cytoplasmic staining was observed in esophageal cells. Ki67 and p53 were defined as positive when nuclear staining was clearly detected at ×10 magnification. FAS expression was reduced in 43% of patients treated with Esomeprazole continuously in comparison to the 10% of patients treated with Esomeprazole on demand (*p* = 0.002). Ki67 expression was reduced in 28% of continuously treated patients in comparison to 5% of patients treated on demand (*p* = 0.001). The p53 expression decreased in 19% of continuously treated patients in comparison to an increase in 2 patients (9%) treated on demand (*p* = 0.05). Continuously Esomeprazole treatment could help in the diminution of metabolic and proliferative activities in the esophageal columnar epithelium and in part it can help prevent the oxidative damage against cellular DNA, resulting in a diminution in p53 expression.

## 1. Background

Barrett’s esophagus (BE) is a lesion characterized by the replacement of normal squamous epithelium with intestinal-type columnar epithelium [1]. This process is induced by an inflammatory process linked to prolonged exposure to gastric acids.

Typical of chronic gastroesophageal reflux disease (GERD), the percentage of patients affected by GERD who will fall ill with this pathology varies according to current cases from 5% to 12% [2]. Currently, BE is considered the only known precancerous disease that precedes dysplasia and therefore the onset of esophageal adenocarcinoma (EAC). EAC is a malignancy with an increasing incidence in all industrialized countries. [3]. It has been demonstrated with surveillance studies that adenocarcinomas develop from a multistep morphological pathway but the proportions of patients in BE follow-up, that evidence a transition from metaplasia to dysplasia is still uncertain [4,5,6]. Dysplasia initiation is the key event for progression to malignancy of a pre-cancerous lesion, defined by Riddell et al. [7] as an unequivocal neoplastic epithelium strictly confined to the basement membrane of the gland from which it arises. Several literature data [8,9,10] suggested that chronic reflux is the main cause of BE, replacing with columnar–cell metaplasia the native squamous–cell epithelium of the esophageal mucosa. Patients with BE have the highest esophageal acid exposure profile among the different gastroesophageal reflux disease (GERD) groups and, additionally, these patients have an impaired sensitivity to esophageal experimental acid perfusion compared with patients with uncomplicated GERD [10,11], healthy subject and patients with reflux symptoms after partial gastrectomy [12]. 

Champion et al. [12] observed that in patients with BE reflux duodenal contents, measured by bilirubin absorbance, are increased, and demonstrated that both acid and duodenal contents, which frequently occur simultaneously in most reflux episodes, worsened the grade of severity of mucosal lesions [13,14]. The literature contains conflicting data regarding the possibility that treatment with agents that inhibit gastric acid secretion significantly reduces the risk of EAC [15,16,17,18]. Indeed, in the last 20 years, it would seem that these drugs have not stopped the incidence of this tumor [15]. According to the opinion of Lagergreen et al. [15], medical or surgical treatment of BE would not serve to prevent EAC but could be useful in preventing the development of irreversible pre-cancerous lesions.to reduce inflammation, the potential chronic stimulus to proliferation. Contrarily, preclinical (biomarker-based) and some observational studies have shown that PPIs may prevent neoplastic progression in patients with BE supporting their role as chemopreventive agents [16,17,18].

For these reasons, this study aimed to test markers of cell proliferation (Ki67, p-53) useful in the routine immuno-histochemical evaluation of esophageal biopsies [19,20] and cellular metabolic markers such as fatty acid synthase (FAS) [21,22,23,24,25].

Ki67 has been used for a long time as a marker of proliferation in the diagnosis of esophageal pathology, but there is a significant overlapping in Ki67 staining between different lesion groups, which are defined by their histological severity [26,27]. 

The prevalence of p53 gene mutations is very rare in the esophageal mucosa with BE or with low-grade dysplasia (LGD) while it increases in high-grade dysplasia (HGD) and in AEC, ranging from 60 to 80% depending on the cases of cases [19,28,29]. Especially in HGD, an elevated risk of EAC was observed in all cases in which p53 was overexpressed [30].

Fatty acid synthase (FAS) is a 520,000 kd multifunctional, dimeric enzyme possessing seven catalytic domains with an active 4′-phosphopantetheine prosthetic group. FAS has the task of synthesizing long-chain fatty acids starting from acetyl-coenzyme A, from malonyl-coenzyme A, which derives from nicotinamide adenine dinucleotide phosphate (NADPH), and from adenosine triphosphate. The end products of FAS activity are myristate, stearate, and palmitate [23]. It has been observed that this enzyme, normally inactive, is found in hormone-sensitive tumors capable of eliciting the biosynthesis of fatty acids [24,25]. One study [31] demonstrated that inhibition of FAS induces apoptosis in tumor cells. This would find justification in the fact that fatty acids are an essential constituent of membrane phospholipids and therefore, their unavailability would seem to limit aberrant cell proliferation [31]. FAS is expressed in many human cancers [32,33,34,35,36,37,38,39] and is differently expressed along the esophagitis-intestinal metaplasia-adenocarcinoma sequence [25]. To intercept the evolution of a BE it is important to stratify patients into selected prognostic subgroups in order to obtain better therapeutic approaches [25]. The purpose of ours was to evaluate if, after one year of continuous or discontinuous treatment with Esomeprazole 40 mg/day in two groups of BE patients, there is a difference in proliferation and metabolic cellular activities in comparison to the initial expression. 

## 2. Patients and Methods

### 2.1. Patients 

We considered a group of consecutive dyspeptic patients prospectively evaluated clinically and with endoscopic and histological examinations. Of these, 42 patients were diagnosed with intestinal metaplasia (Barrett’s metaplasia), 19 females and 23 males, with a mean age of 50.44 ± 10.16, were followed up for one year after the diagnosis and included in the study. 

The inclusion criteria for this study included being over 18 years of age, a duration of at least one year of dyspeptic symptoms which included typical symptoms such as heartburn (a burning sensation rising from the stomach or lower chest to the neck) at least three episodes a week; diagnosis of other esophageal pathology in other previous endoscopic examinations. Finally, the study does not include dyspeptic patients with renal failure and electrolyte imbalance [40].

Patients on active nonsteroidal anti-inflammatory drug (NSAID) treatment were excluded; Those who have a history of previous surgery of the upper gastrointestinal tract; if they had after-effects of gastric complications related to peptic disease. Patients diagnosed with scleroderma, diabetes mellitus associated with autonomic or peripheral neuropathy, myopathy, or the presence of any condition that could affect esophageal sphincter function or esophageal clearing function were excluded from the analysis. Patients who previous to the inclusion in the study used drugs on demand such as antacids, or alginic acid apart from the prescribed dose of proton-pump inhibitors, were excluded from the study.

**Figure 1:**
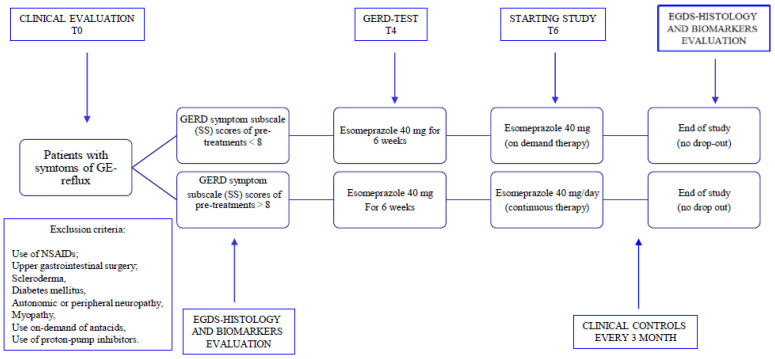
Design of the study.

After endoscopic and histological diagnosis, patients were randomized into two different groups, using a computerized randomized schedule. The first group included patients with a prescription for one year of continuous Esomeprazole 40 mg/day therapy (group A). The second group included patients who assumed discontinuously Esomeprazole 40 mg/day therapy (four-week of Esomeprazole 40 mg-based regimens assumed every three months, coinciding with the change of seasons and in the other periods on-demand in case of heartburn and/or regurgitation (group B) (Figure 1).

Patients were followed-up with clinical controls every three months. Symptom questions have been made regarding epigastric pain and heartburn, to acid regurgitation. Symptom severity was assessed and quantified before and after 4 weeks of treatment using the Gastroesophageal Reflux and Dyspepsia Therapeutic Efficacy and Satisfaction Test (GERD-TEST). Depending on the result obtained, the patients were divided into two groups [41,42] (Figure 1). Informed consent, both written and oral, was obtained from each participant first entering the follow-up phase. Being an observational study, using a drug that has already been used for a long time with rather varied treatment schemes but without involving additional risks. In our case, the ethics committee was made aware of the study and did not deem it necessary to express an opinion on the type of study.

### 2.2. Endoscopy 

Patients eligible for the study entered the follow-up and initially performed an esophagogastroduodenoscopy with multiple endoscopic sampling, in order to evaluate the presence of lesions of the esophageal and gastric mucosa and the search for H. pylori. The severity of esophageal inflammation was assessed using the Los Angeles classification as follows [43]: normal mucosa (no abnormalities); grade A, one (or more) erosions of height extension less than 5 mm; grade B, one (or more) mucosal erosions more than 5 mm in height; grade C, one (or more) continuous mucosal erosions involving up to 75% of the circumference of the distal esophagus lumen; grade D, one (or more) mucosal erosions involving more than 75% of the esophageal circumference, and complicated by lesions. BE was defined as the presence of intestinal metaplasia on biopsy with a length of at least 3 cm, determined by measuring it from the proximal margin of the columnar epithelium to the end of the tubular esophagus or the proximal margin of the mucosal folds in the case of hiatal hernia [44]. Within the lesion, multiple biopsies were taken in all four quadrants at 1-cm intervals according to the Seattle biopsy protocol [45].

After the follow-up period, patients underwent the last endoscopic control to assess, according to Los Angeles criteria [43], the entity of esophageal mucosal damage after the two different Esomeprazole 40 mg-based regimens. BE was classified according to the recommendations regarding the diagnosis and management of BE [44]. In particular, BE patients underwent biopsies of the epithelium in all four quadrants at a distance of 1 cm along the ectopic area [45]. All samples were stored in a solution containing 10% buffered formalin and subsequently embedded in paraffin. Subsequently, they were subjected to staining based on hematoxylin and eosin combined with an acid pH solution based on alcian blue (pH 2.5).

### 2.3. Histology

The bioptic samples taken during the endoscopic examination were by two expert anatomical pathologists. Histological specimens were first prepared and then stained with hematoxylin-eosin and alcian blue. The histological findings were classified by the two experts according to morphological criteria modified by Richardson et al. [46]. The possible presence of dysplasia in the samples was analyzed and its classification was performed according to Geboes’ criteria [47].

### 2.4. Determination of FAS Expression

To determine the degree of FAS expression, BE biopsy specimens were placed on glass slides and incubated with the primary FAS-specific antibody at a concentration of 1:3000 (LSAB2, Dako Corporation, Carpenteria, CA, USA) for the duration of one night. Subsequently, the slides were placed in a reagent containing avidin-biotin and a chromogen 3,3′-diaminobenzidine. Nuclear counterstaining was achieved using Meyer’s hematoxylin. Subsequently, the slides ready for viewing were semi-quantitatively judged for FAS expression. The final judgment of granular cytoplasmic staining in cells was determined by the agreement of the opinion of two blinded pathologists.

### 2.5. Determination of Ki67 Expression

Sections of biopsy specimens arranged in series were first stained with hematoxylin/eosin. An immuno-peroxidase-based preparatory technique was used to obtain the specific staining for Ki67. The sections thus pre-treated were prepared on pre-cleaned coated slides and brought to a temperature of 56 °C for 60 min. Subsequently, the histological sections were deparaffinized with xylene and rehydrated in a solution with distilled water. Recovery of the epitope produced by heat exposure was performed in boiling 0.01 citrate buffer at pH 6.0 for 15 min. The sections were subsequently incubated for 40 min with anti-Ki67 diluted 1:50 (MIB-1; Beckman Coulter, Fullerton, CA, USA) and immersed in avidin-biotin and diaminobenzidine tetrahydrochloride. Immunoreactivity was subsequently evaluated by two blinded pathologists.

### 2.6. Determination of p53 Expression

Immunohistochemistry for p53 determination was performed starting from formalin-fixed, paraffin-embedded biopsy specimens of the esophageal mucosa. The blocks thus obtained were sectioned in order to obtain sections of 3 µm. These sections were mounted on Super-Frost/Plus microscope slides (BDH Laboratory Supplies, Menzel, Germany). All slides were subsequently deparaffinized in xylene and then rehydrated in ethanol. Then for 15 min, the rehydrated samples were treated with a methanol-hydrogen peroxide solution. Subsequently, the sections were treated with the anti-p53 monoclonal antibody DO 7 (dilution 1:500), and the anti-p53 polyclonal antibody CM-1 (dilution 1:250) were used in order to obtain the expression intranuclear cell of p53. A semiquantitative assessment of p53 immunoreactivity was subsequently performed by two blinded pathologists.

### 2.7. Determination and Evaluation of the Degree of p53, Ki67, and FAS Expression

The staining of the Ki67 nuclear level and FAS levels in the cell cytoplasm were classified as negative (no immunoreactivity); mildly positive (staining in less than 50% of epithelial cells); highly positive (staining in more than 50% of epithelial cells) [25]. The determination of p53 expression was evaluated based on the reactivity of the nuclei expressed as a percentage value and classified with an intensity scale from 0 to 3, with 0+ representing no staining; 1+ light staining; 2+ moderate coloration; 3+ representing intense coloration [48].

### 2.8. Statistical Analysis

Pearson’s Chi-square test was used to compare the immunohistochemical expression of each antibody, FAS (cytoplasmic metabolic cellular marker), and p53 and Ki67, nuclear markers in the group of Esomeprazole continuously-treated patients (group A) in comparison to the group of on-demand Esomeprazole–treated patients (group B). A value of *p* < 0.05 was considered significant.

## 3. Results

### 3.1. Patient’s Characteristics

A total of 42 BE patients were enrolled in the study. Male and female were 23 and 19 respectively with a mean age of 55.5 yrs ± 13 (Table 1). At enrollment, 8 patients referred heartburn only, 5 patients referred regurgitation only, and 29 patients referred heartburn and regurgitation combined. Nightly symptoms were present in 36 patients. No patients presented with symptoms once a week, 21 patients 6 times a week, and 21 presented with symptoms at least 15 times or more than 15 times a week.

At endoscopy, patients with BE showed erosions and metaplastic area with a mean extension of 2.8 ± 0.6 cm (cm). Histological examination showed a concordant diagnosis of intestinal metaplasia. No patients showed specimen dysplasia in the biopsy.

After a period of Esomeprazole 40 mg day therapy of 6 weeks, on the basis of study design, patients with symptoms occurrence of 15 times per week were assigned to continue the same treatment for one year, while patients with symptoms frequency of 6 times per week were treated with Esomeprazole 40 mg/day on demand for a period of one year. In group A, BE showed an extension of 2.8 ± 0.9 cm while in group B 2.8 ± 0.7 cm (Table 1).

At the end of the study, all patients of group A presented with a reduction in symptoms (18 experimented with symptoms once a week and 3 six times a week), while in patients of group B 12 patients reported a diminution of symptoms, but in minor part comparison to group A (15 patients referred symptoms at least 6 times per week and 6 patients referred the persistence of symptoms until 15 times per week. At endoscopy, after one year’s follow-up, the mean extension of BE was reduced to 2.55 ± 0.4, and in particular, was 2.5 ± 0.7 in patients with group A and 2.6 ± 0.1, *p* = ns.

### 3.2. Immunohistochemical Evaluation 

#### 3.2.1. FAS 

**Figure 2:**
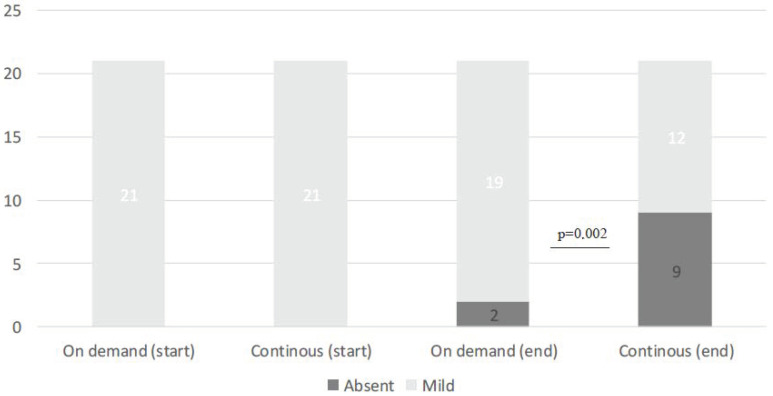
Variation in FAS expression in the two groups.

**Figure 3:**
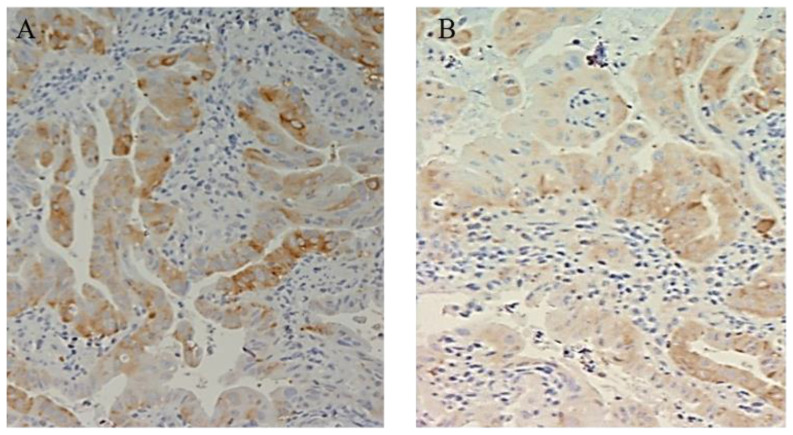
(**A**) FAS expression in patients with BE (cytoplasmic staining 50% of cells recognizable at 10×) at baseline (**B**) FAS Barrett esophagus expression con continuously treated with esomeprazole: (nuclear staining recognizable at 10× magnification in more than 50% of positive cells).

At entry, the percentage of mild positive cytoplasmic FAS expression was present in all (100%) BE patients. A total of 21 patients with positive FAS expression were assigned to group A and 21 were assigned to group B (Table 2). At the end of treatment, FAS expression was reduced in 9 (43%) patients continuously treated with Esomeprazole, in comparison to the reduction that was documented in only 2 patients (10%) out of the 21 treated with Esomeprazole on demand (Pearson chi-square: 16.16; df: 2; *p* = 0.002). None of the patients presented with an increase in cytoplasmic expression of FAS during the follow-up period (Table 3) (Figure 2 and Figure 3).

#### 3.2.2. Ki67

**Figure 4:**
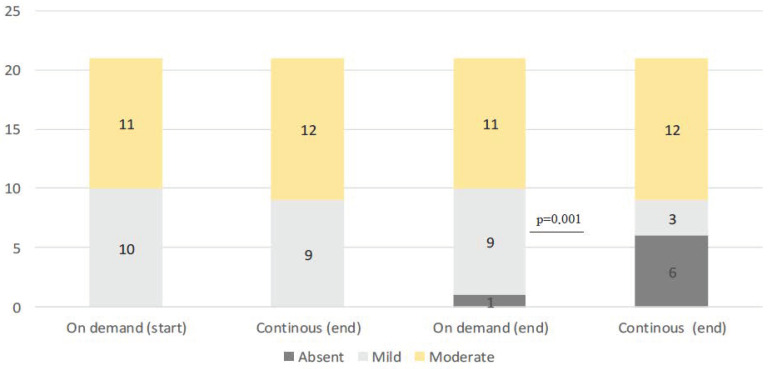
Variation in Ki67 expression in the two groups.

**Figure 5:**
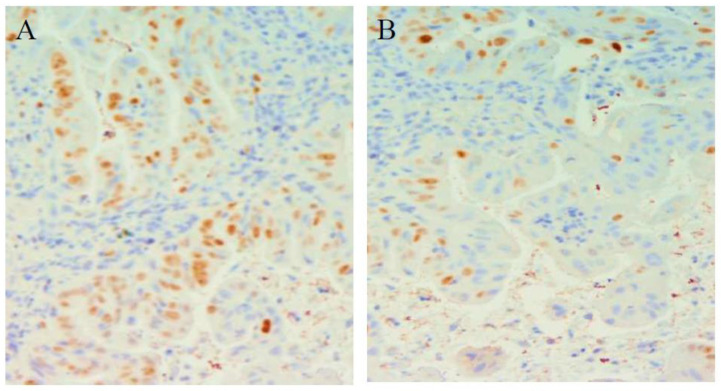
(**A**) Ki67 expression in patients continuously treated with esomeprazole (nuclear staining recognizable at 10× magnification. (**B**) Diminution of Ki67 in Barret’s esophagus (nuclear staining recognizable at 10× magnification in less than 50% of positive cells.

At entry, in patients with BE, mild expression was present in 19 patients (46%) while it was moderate in the other 23 cases. Nine patients with mild expression were assigned to group A and 10 to group B, while of those with moderate expression 12 were assigned to group A and 11 to group B (Table 2). At the final check, Ki67 expression was reduced in 6 (28%) of patients treated continuously versus only one patient (5%) of patients treated on demand (Pearson chi-square: 8.8; fd: 1; *p* = 0.001) (Table 3) (Figure 4 and Figure 5). No patients experienced increased nuclear Ki67 expression during the follow-up period.

#### 3.2.3. p53

**Figure 6:**
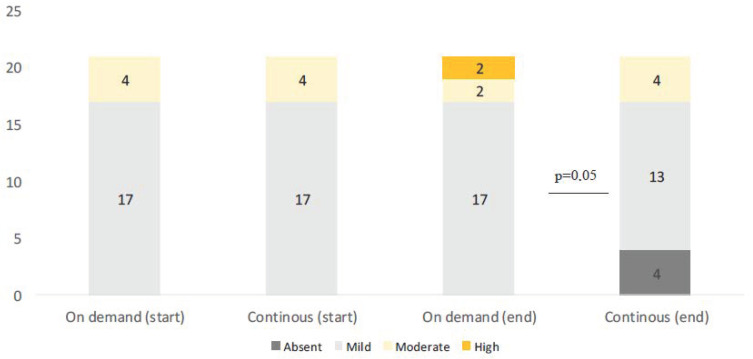
Variation in p53 expression in the two groups.

**Figure 7:**
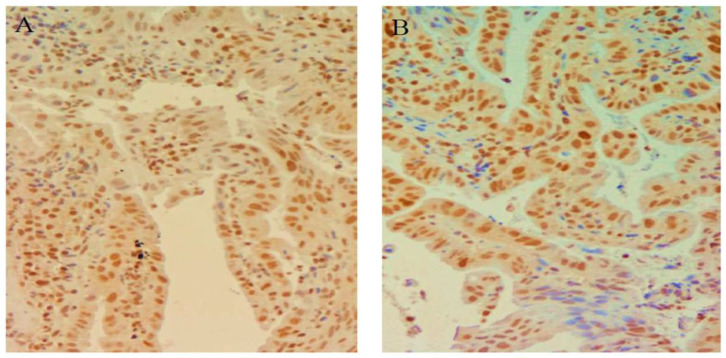
(**A**) p53 expression in patients with Barrett’s esophagus continuously treated. (**B**) p53 expression in patients with Barret’s treated on demand.

At entry, p53 was expressed mildly (1+) in 34 BE patients (80%) and moderate (2+) in 8 BE patients (20%). At randomization, patients were equally divided into both groups (Table 2). At the end of the follow-up period, only 4 patients (all with mild expression) from group A showed a decrease in nuclear p53 expression and none of the patients of group B experimented with an improvement in p53 expression. In contrast, in patients with moderate activity (2+), p53 expression was increased in 2 patients treated on demand (50%) (Pearson chi-square: 15.00; df: 1; *p* = 0.05) (Table 3) (Figure 6 and Figure 7).

## 4. Discussion 

Although esophageal intestinal metaplasia and adenocarcinoma are strictly associated, the overall risk of cancer in BE patients is estimated to be approximately 0.5% per year, and the risk of neoplasia including HGD to be 1.3% per year [49,50,51]. The aim of this study was to evaluate if, after one year of treatment, continuous or discontinuous administration of Esomeprazole 40 mg/day affected the expression of proliferation and metabolic cellular markers activities in comparison to the initial expression, in two groups of BE patients. The present study showed that, after one year of Esomeprazole treatment, p53 remained over-expressed and worsened especially in patients with discontinuous treatment with Esomeprazole. BE is a tissue metaplasia that usually develops in patients with chronic damage to the esophageal mucosa caused by gastroesophageal reflux disease (GERD). However, it is unclear how the mucosa consisting of columnar epithelium replaces the squamous mucosa upon exposure to gastric contents [52]. The two most accredited hypotheses to explain the differentiation between squamous epithelium and columnar epithelium are trans-differentiation and trans-commitment [52]. These hypotheses are complementary to each other and are not mutually exclusive, much less the hypothesis of a single progenitor cell which, in the context of the basal layer of the mucosa, leads to the phenotypic mutation of these cells [53,54]. Thus, in the latter case, the BE formation process is not dissimilar from normal wound healing processes, only that this process, under the action of gastric contents, leads the cells to be exposed to chronic damage [53,54]. If chronic reflux is the root cause of BE, there are good enough reasons to consider that gastric contents play an important role in the development of esophageal adenocarcinoma across the various degrees of dysplasia. In the literature, we have found no indication that the treatment of reflux, either continuous or on-demand, reduces the expression of differentiation markers or metabolic activity markers of these. Furthermore, the effect of long-term treatment with a proton pump inhibitor is unknown on these patients and its utility still remains a matter of debate. The possible clinical implications to be drawn from our data support the hypothesis that long-term therapy with proton pump inhibitors associated with endoscopic surveillance in patients with symptomatic reflux can be effective if administered before the development of irreversible precancerous changes. Using such therapies and improving molecular screening, there is a theoretical possibility of reducing the number of cases progressing to dysplasia. The search for dysplasia is still considered the best predictor of the malignant progression of BE [55]. Recent advances in biomarker identification have highlighted the potential for improvements in risk stratification [55,56]; however, biomarkers are not consistently included in dysplasia screening methods covered by the major guidelines of the GI societies [55]. 

According to our data, the decrease of proliferation in the columnar epithelium of the esophagus induced by continuous therapy with Esomeprazole could reduce the effect of oxidative damage against cellular DNA as claimed by other studies [57,58], and this also has repercussions in the reduction of the proliferative and metabolic activity in the context of the BE determining a reduction in p53 expression’s, sign of DNA repair systems activation. In fact, continuous treatment with Esomeprazole achieves the best results in improving Ki67 and FAS expressions and is useful in decreasing metabolic and proliferative activities in the esophageal columnar epithelium. It has been hypothesized that lipogenesis is a metabolic process common to all cells in active replication such as inflammatory or neoplastic cells [31]. For this reason, it is possible to use the determination of the degree of expression of FAS to increase the diagnostic power in the context of lesions attributable to BE, considering that it is a key enzyme of anaerobic metabolism which is activated together with aerobic metabolism only to promote replicative activity. FAS is therefore an expression of the metabolic activity of cells in active replication while p53 and Ki67 are markers of this active proliferation also in the esophageal epithelium subjected to damage by gastric acids. of the cell [59,60]. Taking into account the role of acid secretion in esophageal epithelial damage, proton pump inhibitors appear to be actually the only treatment that can interfere with the worsening of histological damage. Indeed, numerous studies have shown that acid-suppressing proton pump inhibitor therapy stabilizes the activity of cellular proliferative cells in the BE and consequently can prevent dysplasia and have a positive impact on reducing the risk of cancer in the esophagus [61,62,63,64,65,66] especially if the therapy is continuous and long-term [67]. The mechanism by which proton pump inhibitors exert a protective effect is nonetheless unclear. In BE, metaplastic cells have a higher proliferative rate and are in line with what has been demonstrated both in vitro and in vivo, in esophageal adenocarcinoma cell lines under acid exposure through the production of agents by mitogens that transmit signals regulating cell growth and proliferation, and contextually decrease apoptosis [64]. Hillmann et al. [67] and other Authors [63,64,65], demonstrated that these drugs stabilize the proliferation of epithelial cells already in the short term suggesting that the prolongation of the duration will limit the hyperplasia of the basal cell layer [68,69]. Our results are in agreement with this hypothesis; we monitored the patients after one year of follow-up by observing a decrease in Ki67 expression, a sign of a decrease in cell proliferation, and contextually a decrease in FAS-mediated supplementary metabolic activities, a sign of loss of cellular metabolic demand. On the other hand, some studies have demonstrated that intermittent acid exposure has antiproliferative effects in non-neoplastic Barrett epithelial cell lines [70,71] and it has been postulated that the effect of PPIs may be mediated by rather anti-inflammatory effects, which has an effect on proliferation [72]. Our study suggests that continuous PPIs administration achieved the best results compared to on-demand or discontinuous administration, in terms of a decrease of FAS and Ki67 expressions and regarding the control of p53 expression, which drastically worsened in discontinuously treated patients. 

FAS is an enzymatic complex capable of synthesizing fatty acids, starting from acetyl-CoA and malonyl-CoA, in the presence of NADPH. It is mainly expressed in the liver and adipose tissue, but low levels are also found in other tissues including the esophagus and colon [25,31]. Its main function is to catalyze the synthesis of palmitate [73]. FAS is the only human enzyme complex responsible for converting dietary carbohydrates into fat. FAS is downregulated in normal cells, while in contrast, several human tumors and actively replicating tissues overexpress the enzyme [73]. This is because, in tissues experiencing active and uncontrolled replication, fatty acids are essential for the production and storage of energy, and for building constituent elements of cellular structures such as membranes and intermediates in the biosynthesis of hormones and other biologically important molecules [74]. FAS metabolism and homeostasis are transcriptionally regulated by upstream stimulatory factors and sterol regulatory element-binding protein-1c (SREBP-1c) [73]. Our study showed that FAS expression decreased significantly in patients treated with continuous anti-secretory therapy compared to those treated on demand and this data was more related to the decrease of p53 than to cell proliferation represented by the expression of Ki67, which was significantly decreased, especially in patients treated continuously with proton pump inhibitors. This means that PPIs are able to partially reduce cell proliferation while they appear to be more active in inducing a reduction in the metabolic demands of BE cells. Furthermore, the high expression rate in the BE patient at the start of the study suggests that such cells behave metabolically, such as malignant cells, and thus have higher metabolic demands than normal cells. The p53 protein is a nuclear transcription factor involved in important cellular functions such as transcription, DNA synthesis, repair, arrest of the cell cycle, aging, and apoptosis [74]. The common feature of all cancer cells is the deregulation of cell proliferation with the consequent loss of the ability to undergo apoptosis [74]. In this context, the role played by the p53 protein in both maintenance and maintenance is fundamental to genomic integrity, and in the suppression of tumor genesis. In physiological conditions and therefore in normal cells, p53 levels are kept low thanks to rapid degradation. The activation of p53 occurs in response to multiple signals of cellular stress, including direct DNA damage, growth factor deprivation, or in response to hypoxia and anoxia [74]. For this role, p53 is important in active tissue replication such as BE. Our study observed a decrease in metabolic activity in tissue samples examined after continuous proton pump inhibitors therapy, consistent with a mild decrease in p53 expression. Instead, in patients treated on request, we noticed an increase in the expression of p53, a sign that such therapy has little effect on the probable dysplastic transformation of the BE. In conclusion, the study highlighted an activation of FAS also in the context of BE, a sign that this lesion possibly entails an increase in cellular metabolic needs comparable to that of tumor cells. The study also highlighted how a continuous suppression of acid production induced by Esomeprazole is able to repress the metabolic demands of cells in the context of BE and is able to reduce the expression of p53, especially in cases where said expression was mild degree. Conversely, discontinuous therapy is not able to contrast the increase in cellular metabolic activity and leads to a worsening of p53 expression, a sign of the persistence of oxidative damage against cellular DNA.

## Figures and Tables

**Table 1:** General characteristics of patients.

Characteristics	Total	Group A	Group B
Sex	18 females and 24 males		
Age	50.44 ± 10.16	51.21 ± 6.6	49.23 ± 4.4
Length of BE at entry	2.8 ± 0.6	2.9 ± 0.2	2.7 ± 0.8
Length of BE at exit	2.55 ± 0.4	2.5 ± 0.7	2.6 ± 0.1
Smokers	16/42	7/42	9/21
Alcohol consumers	7/42	3/21	4/21
Heartburn	8/42	5/21	3/21
Regurgitation	5/42	4/21	4/21
Heartburn and regurgitation	29/42	19/21	10/21
Nightly symptoms	36/42	17/21	15/21
Number of episodes/week < 7	21/42	11/10	10/10
Number of episodes/week > 8	21/42	12/21	9/21

**Table 2:** Frequency of p53, Ki67, and FAS expression in patients with BE randomized to continuous therapy and on-demand therapy.

	Long-Term PPIs	On-Demand PPIs.
*p53*		
Mild	17 (80%)	17 (80%)
Intense	4 (20%)	4 (20%)
*Ki67*		
Mild	9 (43%)	10 (48%)
Moderate	12 (57%)	11(52%)
*FAS*		
Mild	21 (100%)	21 (100%)
Intense	0	0

**Table 3:** Frequency of p53 and FAS Ki67 and FAS expressions in patients with BE at the end of the follow-up period.

	Long-Term PPIs	On-Demand PPIs
*p53*		
Absent Mild	4 (19%) 13 (62%)	0 17 (82%)
Moderate Intense	4 (19%)	2 (9%) 2 (9%)
*Pearson chi-square: 15.00; df: 1; p = 0.05;*		
*FAS*		
Absent	9 (43%)	2 (10%)
Mild	11 (56%)	19 (90%)
Intense	0	0
*Pearson chi-square: 16.16; df: 2; p = 0.002;*		
*i67*		
Absent	6 (28%)	1 (5%)
Mild	3 (14%)	9 (43%)
Moderate	12 (58%)	11 (52%)
*Pearson chi-square: 8.8; fd: 1; p = 0.001*		

## Data Availability

The data that support the findings of this study are available from the corresponding author upon reasonable request.
